# 

**Published:** 1889

**Authors:** 


					﻿DOUBLE NUMBER.
Vol. IX.
October and November, 1889.
Nos. 10 & 11.
THE
HOMEOPATHIC pHY^iClAfl,
A Monthly Journal of Homoeopathic Materia Medica
and Clinical Medicine.
“He is a freeman whom truth makes free, and all are slaves beside."
“Seek the Truth: come whence it may, cost what it will."
EDITED AND PUBLISHED BY
Edmund j. lee, m. D.,
AND
WALTER M. JAMES, M. D.
PHILADELPHIA :
No. 1185 SPRUCE STREET.
LONDON
ALFRED HEATH & CO., Sole Agents foe Great Britain,
114 Ebury Street, Eaton Square.
-Yearly Subscription, $2.SO. invariably in advance. Single numbers, 25 cts.
Single numbers containing the Repertory Supplement, 75 cts.
Entered at the Philadelphia Poet-Office aa Second* Close Mall Matter.
				

